# QT prolongation alerts lead to monitoring but rarely to therapeutic changes: a prospective hospital study

**DOI:** 10.3389/fphar.2026.1833921

**Published:** 2026-05-07

**Authors:** Aurélien Simona, Tiago Lourenço, Christian Lovis, Dina Zekry, Didier Hannouche, Nathalie Vernaz, Caroline Flora Samer

**Affiliations:** 1 Department of Acute Care Medicine, Geneva University Hospitals, Division of Clinical Pharmacology and Toxicology, Geneva, Switzerland; 2 School of Pharmaceutical Sciences, Faculty of Sciences, University of Geneva, Geneva, Switzerland; 3 Division of Medical Information Sciences, University Hospitals of Geneva, Geneva, Switzerland; 4 Department of Radiology and Medical Informatics, Faculty of Medicine, University of Geneva, Geneva, Switzerland; 5 Department of Rehabilitation and Geriatrics, Division of Internal Medicine for the Aged, Geneva University Hospitals, Geneva, Switzerland; 6 Department of Orthopaedic and Trauma Surgery, Geneva University Hospitals, Geneva, Switzerland; 7 Medical Directorate, Finance Directorate, Geneva University Hospitals, Geneva, Switzerland; 8 Department of Anaesthesiology, Pharmacology, Intensive Care and Emergency Medicine, Faculty of Medicine, University of Geneva, Geneva, Switzerland

**Keywords:** alert fatigue, clinical decision support system, medication safety, patient safety, pharmacodynamic alerts, QT prolongation

## Abstract

**Introduction:**

Drug-induced QT interval prolongation is a well-recognized safety concern in hospitalized patients. Clinical decision support systems (CDSS) generate alerts to identify high-risk drug combinations, but their impact on clinical management remains uncertain.

**Methods:**

This study evaluated clinical responses from prescribing clinicians to QT prolongation alerts, focusing on electrocardiogram (ECG) monitoring and treatment modifications. We conducted a 10-month prospective study in two hospital wards (acute geriatrics and orthopedics) at Geneva University Hospitals. The CDSS embedded within the electronic health record used a pharmacodynamic risk scoring system (Riskbase) that assigns scores to individual drugs based on pharmacological and clinical evidence and generates alerts when cumulative risk exceeds a predefined threshold. Clinical interventions were assessed within 7 days following high-risk QT alerts.

**Results:**

A total of 154 patients were included (123 in geriatrics and 31 in orthopedics). Intervention rates ranged from 60.2% to 91.9% in geriatrics and from 45.2% to 80.6% in orthopedics, depending on the time window. However, these interventions were almost exclusively driven by ECG monitoring. Treatment modifications were rare, occurring in only 2.0% of patients. Most alerts were associated with combinations of low- or moderate-risk drugs, particularly antipsychotics, antidepressants, and antiemetics, some of which may have alternatives with lower QT-prolongation potential and may be considered depending on the clinical context. In addition, medications prescribed on an as-needed basis contributed substantially to QT risk scores, despite limited recent administration.

**Discussion:**

In clinical practice, QT prolongation alerts are associated with frequent monitoring but rarely lead to therapeutic changes. These findings highlight a gap between risk identification and pharmacological risk mitigation and suggest opportunities to improve medication safety through more targeted prescribing and optimization of alert systems.

## 1 Introduction

Long QT syndrome is a type A adverse drug reaction (ADRs), resulting from dose-dependent effects on cardiac ion channels. QT interval prolongation, measured on an electrocardiogram (ECG) and corrected for heart rate (QTc), increases the risk of torsades de pointes (TdP) and sudden cardiac death. QTc prolongation thresholds vary across guidelines. Traditional cut-offs define prolongation as QTc >450 ms in men and >460 ms in women (Kotsialou et al., 2024; Davies et al., 2023), while recent European Society of Cardiology (ESC) guidelines recommend gender-independent thresholds: QTc ≥480 ms, or ≥460 ms in symptomatic individuals (Zeppenfeld et al., 2022). While TdP itself remains a rare event in hospitalized patients, the risk significantly increases when QTc exceeds 500 m or rises by more than 60 m from baseline (Tisdale et al., 2020).

Multiple factors predispose patients to QT prolongation, including advanced age, female sex, electrolyte disturbances, cardiovascular disease, bradycardia, and drug–drug interactions (Vandael et al., 2017; Wiśniowska et al., 2016). QT-prolonging drugs are widely used across therapeutic classes such as antipsychotics, antidepressants, and antiemetics, making drug-induced QT prolongation a common safety concern in hospitalized patients.

Given the potential for severe adverse outcomes, clinical decision support systems (CDSS) have been implemented to generate automated alerts for high-risk drug combinations (Tisdale et al., 2014). However, some studies have suggested that such alerts may not only fail to improve clinical outcomes but could even be associated with worse outcomes, such as increased mortality, although these findings remain inconclusive (Trinkley et al., 2025). In addition, a growing body of evidence suggests that drug safety alerts are frequently ignored or overridden by clinicians. Alert fatigue, a phenomenon where an excessive number of system-generated warnings leads to desensitization and reduced compliance, has been identified as a key challenge in the effectiveness of CDSS interventions (van der Sijs et al., 2006; Baysari et al., 2017).

This study seeks to assess whether pharmacodynamic drug safety alerts for QT prolongation are followed by clinical interventions, specifically ECG monitoring and/or treatment modification. By analyzing data from QT prolongation alerts in a hospital setting, this research aims to provide insights into current prescribing practices and identify opportunities for improving patient safety and reducing alert fatigue.

## 2 Materials and methods

### 2.1 Study setting

This prospective interventional study was conducted at Geneva University Hospitals (HUG) over a 10-month period from July 2020 to April 2021. The study focused on two medical wards—acute geriatrics care and orthopedics—chosen for their distinct patient populations with varying comorbidities and prescribing practices.

### 2.2 QT prolongation data source

The electronic health records at the HUG integrate computerized physician order entry (CPOE) with a CDSS based on a commercial knowledge database (Riskbase) provided by Medbase, which is a company that provides comprehensive drug information and clinical decision support (Medbase, 2026). Riskbase draws on pharmacological and clinical expertise from academic and clinical environments, with origins in institutions such as the Karolinska Institutet in Sweden and collaborations with clinical pharmacology and healthcare services in Finland (Böttiger et al., 2018). However, detailed information regarding its underlying data sources and development process is not fully publicly available due to its proprietary nature. The database used in this study for QT prolongation flags risk levels of each medication. Around 1800 substances have been associated with a score based on clinical and preclinical data on adverse effects, along with clinical and preclinical evidence regarding receptor affinity. The scores range from 0 (no pharmacological effect) to 3 (strong QT-prolonging effect). The CDSS calculates a cumulative QT risk score by summing the individual scores of all prescribed drugs. Alerts are categorized into four levels: no risk (category A), low risk (category B), moderate risk (category C), and high risk (category D). High risk of QT prolongation is defined by a cumulative score ≥4. This threshold is predefined within the CDSS and was not determined by the investigators. For example, the total QT prolongation score for concomitantly prescribed amiodarone (individual score = 3) and haloperidol (individual score = 2) has a total score of 5, thereby classifying this prescription as high risk for QT interval prolongation since the total score exceeds 4.

To provide contextual comparison with an established reference, a descriptive mapping between Riskbase classifications and CredibleMeds categories was performed for QT-prolonging drugs involved in the alerts observed in our study, rather than for all drugs listed in either database. CredibleMeds is a widely used, evidence-based resource that classifies drugs according to their risk of TdP into categories such as ‘known’, ‘possible’, and ‘conditional’ risk, based on clinical, pharmacovigilance, and regulatory data. It is commonly used in clinical practice and research as a reference standard for QT risk assessment. This comparison was intended to provide external context and support the interpretability of the Riskbase classification (Crediblemeds, 2025). This comparison was based on drug-level classification and is presented in the Supplementary Material.

### 2.3 CDSS design

**FIGURE 1:**
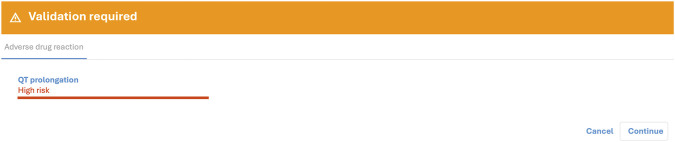
High-risk alert actively triggered during prescription validation.

Pharmacodynamic alerts were triggered both passively and actively during prescribing depending on QT prolongation risk category and were routinely logged for review:

- Risk categories and risk scores (all categories, from no risk to high risk) for all pharmacodynamic alerts (including QT prolongation) were **passively** available at any time by clicking an icon in the prescription screen toolbar.
- During prescription validation (i.e., electronic signature), only high-risk (category D) alerts **actively** interrupted the prescription process (Figure 1), offering two choices: “continue” or “cancel” on the prescription screen.

Alerts were displayed to prescribing clinicians during the electronic prescription validation process.

### 2.4 Study population

Between July 2020 and April 2021, 154 patients with overridden QTc prolongation alerts triggered during prescription validation were considered. Patient exclusion criteria included outpatients, ventricular pacemaker use, and lack of structured electronic prescriptions.

### 2.5 Data collection

Alert instances were identified through the CDSS log files, which systematically recorded every warning triggered at the time of prescription, regardless of the physician’s subsequent interaction with the alert (e.g., overriding or closing the window). For each logged alert, data on ECGs, risk factors for QT prolongation, and drug prescriptions were extracted from electronic health records (EHRs) for manual review. The study analyzed the clinical intervention rate (ECG recording and/or treatment modification) within 1, 3, 5, and 7 days after a high-risk long QT alert was actively displayed. These time windows were selected *a priori* to capture short-term clinical responses while allowing sufficient time for clinicians to act. The 7-day interval represents a pragmatic upper bound within a clinically relevant timeframe and is broadly consistent with the time course over which many drugs approach steady state, although this varies across medications (DeVane, 1994).

The following non-pharmacological risk factors for QT prolongation were assessed: age >65 years, female sex, impaired renal function, impaired liver function, bradycardia, cardiovascular disease, hypothyroidism, hypokalemia, hypocalcemia, and hypomagnesemia. The variables related to comorbidities (e.g., impaired liver function) were identified based on routinely available clinical data within the electronic health record, including codes from the 10th revision of the International Classification of Diseases (ICD-10 codes) and information extracted from free-text clinical documentation. The selection of risk factors was guided by established literature on QT prolongation risk and, importantly, by the availability and reliability of data within the EHR. The factors included represent those that could be consistently and feasibly extracted within the scope of this study. Other relevant variables, such as substance use or chronic inflammatory conditions, would have required substantially more complex and resource-intensive data collection processes, and were not systematically available in the dataset.

### 2.6 Outcomes

The primary composite outcome was defined as the rate of clinical intervention following a first high-risk QT prolongation alert (category D, cumulative score ≥4) for a patient. Clinical intervention was defined as the performance of an ECG and/or modification of the prescribed QT-prolonging medication leading to lower risk category (category A-C, cumulative score <4). Secondarily, we aimed at characterizing the distribution of QT-prolonging medications prescribed at the time of alert firing and prevalence of patient risk factors.

### 2.7 QT prolongation algorithms

After the first occurrence of a high-risk long QT alert, a manual chart review was performed by a clinical pharmacologist and a pharmacist, including medication review, verification of ECG completion, and identification of patient risk factors. The rationale defining the primary outcome criteria was based on four long QT management algorithms to ensure that our definition of clinical intervention was consistent with different guideline-based approaches and to increase the robustness of our outcome definition. The first algorithm was published in the *European Heart Journal* by Fanoe et al., in 2014 (Fanoe et al., 2014), the second algorithm comes from the 2022 ESC guideline on cardio-oncology (Lyon et al., 2022), the third algorithm comes from an article published in 2021 in the *Postgraduate Medical Journal* (Khatib et al., 2021), and finally the fourth long QT management algorithm was published by the Canadian Cardiovascular Society in 2023 (Davies et al., 2023).

### 2.8 Statistical analysis

Data cleaning, descriptive statistics and plot rendering were performed using R (version 4.3.1) and RStudio (version 2023.06.1) (R Core Team, 2024). Data manipulation was primarily handled with the tidyverse ecosystem (Wickham et al., 2019), while plots were generated using ggplot2 and its extensions (patchwork, cowplot, ggtext, and ggpubr) for more complex layouts and formatting. To assess whether there was a statistically significant difference in the proportion of intervention rate within 7 days between the geriatric and orthopedic wards, a Fisher exact test of independence was performed since expected values for non-intervention at 7 days in orthopedics were less than 5. A p-value <0.05 was considered as statistically significant.

## 3 Results

Over the study period, 2,730 alerts involved at least one QT-prolonging drug. A total of 1,449 high-risk alerts (long QT risk score ≥4) were displayed.

### 3.1 Clinical intervention rate

The primary composite outcome (ECG and/or treatment modification) was achieved in 60.2%–91.9% of geriatric patients and 45.2%–80.6% of orthopedic patients following a first high-risk QT prolongation alert, within 1-day and 7-day thresholds respectively (Table 1). However, this composite outcome was almost entirely driven by ECG performance. Treatment modifications were rare: only 3 medication changes were observed among the 154 patients (2.0%), all occurring in the geriatric ward. These included a switch from levofloxacin to ceftriaxone, a dose reduction of citalopram, and a temporary discontinuation of quetiapine. In all three cases, the QT interval was measured after the alert was triggered and revealed prolonged intervals ranging from 506 m to 563 m, which subsequently motivated the treatment modifications. These specific ECGs were performed in response to concerns regarding QT prolongation following the CDSS alert. Since these patients had already received an ECG, the subsequent medication changes did not increase the overall intervention rate. Consequently, the intervention rates presented in Table 1 essentially reflect ECG performance alone, with medication optimization playing a negligible role in the clinical response to alerts.

**TABLE 1:** Clinical intervention rate according to day threshold and medical ward.

Threshold	Geriatrics (n = 123)		Orthopedic surgery (n = 31)	
	n	%	n	%
1 day	74	60.2	14	45.2
3 days	97	78.9	18	58.1
5 days	106	86.1	22	71.0
7 days	113	91.9	25	80.6

Intervention count (n) and rate (%) was based on the performance of an ECG, and/or modification of the prescribed QT-prolonging medication leading to lower risk category within the corresponding time window threshold.

In addition, only 5 ECGs (3%) explicitly documented QT prolongation risk as the indication, whereas in 66 cases (43%) no reason for ECG ordering was reported. This lack of documentation complicates the assessment of whether ECGs were truly performed in response to CDSS alerts. Finally, there was a trend toward more frequent intervention in geriatrics (91.6% vs. 80.6%), but the difference was not statistically significant (p = 0.094).

### 3.2 Drug distribution

**FIGURE 2:**
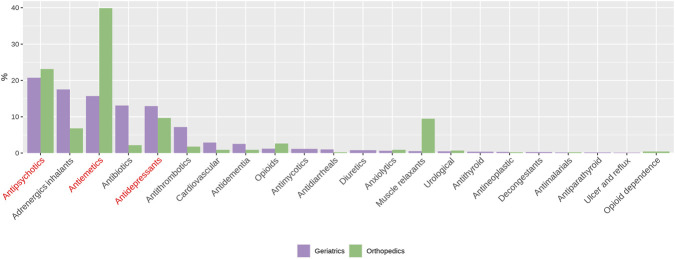
Count distribution of drug classes across geriatric (purple bars) and orthopedic (green bars) wards. Drug classes highlighted in red (antipsychotics, antidepressants, and antiemetics) represent frequently used drug classes for which alternatives within the same class with lower QT-prolongation potential may be considered depending on the clinical context. Drugs have been classified according to ATC codes.

58 drugs were involved in alert firings with 36 showing low-risk of QT prolongation (score 1), 18 moderate-risk (score 2), and 4 high-risk (score 3) drugs (Supplementary Material). Drug classes that were frequently associated with QT prolongation alerts in both medical wards included antipsychotics, antiemetics, and antidepressants (Figure 2).

**FIGURE 3:**
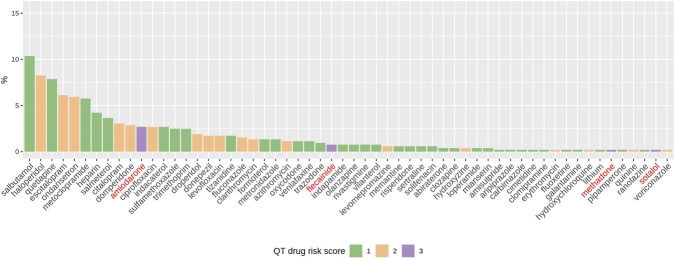
Frequency distribution of drug INNs with severity scores. 58 drugs (displayed by descending order of frequency in geriatrics) were involved in 154 long QT alerts. Green bars = risk score of 1 (36 drugs), orange bars = risk score of 2 (18 drugs), and purple bars = risk score of 3 (4 drugs). Drugs highlighted in red correspond to molecules with the highest QT prolongation risk (score 3).

Drugs carrying high risk of QT prolongation (Figure 3, purple bars) like antiarrhythmic molecules and methadone were involved in less than 4% alert firings, meaning that concomitant low- and moderate-risk drugs (Figure 3, green bars and orange bars respectively) were mainly responsible for increasing the risk of prolonging the QT interval. Among them, frequently used medication like haloperidol (antipsychotic), citalopram/escitalopram (antidepressants), and ondansetron/metoclopramide (antiemetics) were common alert triggering drugs.

**FIGURE 4:**
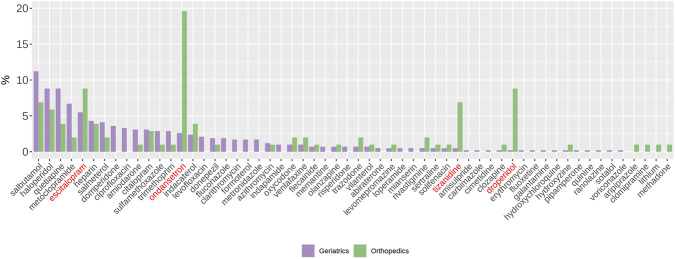
Frequency distribution of drug INNs in geriatrics and orthopedics. Red labels show the top four drugs in orthopedics associated with high risk of QT prolongation.

Also, while various molecules contributed to long QT alerts in geriatric patients, ondansetron (19.6%), escitalopram (8.8%), droperidol (8.8%) and tizanidine (6.9%) in orthopedic patients represented the main source of drug-induced QT prolongation alerts (Figure 4, red labels).

### 3.3 Dosing regimen

**FIGURE 5:**
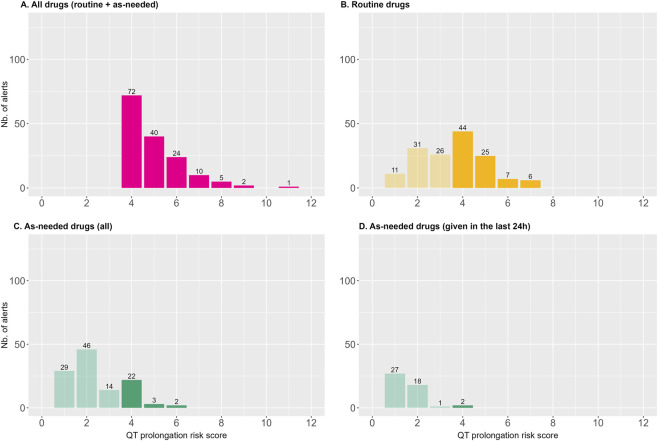
Relative weight of routine and as-needed drugs in the QT prolongation risk score. Number of alerts displayed (y-axis) versus cumulative QT prolongation risk scores (x-axis), where the score corresponds to the sum of individual QT-prolongation risk scores for all prescribed drugs. This is shown for: the full prescription (upper left, **(A)**), routine drugs only (upper right, **(B)**), all as-needed drugs (bottom left, **(C)**), and as-needed drugs administered within 24 h before an alert was triggered (bottom right, **(D)**). Light bars indicate non–high-risk scores (score <4); opaque bars indicate high-risk scores (score ≥4).

The full prescription (i.e., both routine and as-needed drugs) was included in the calculation of the long QT total risk score during prescription validation. Figure 5 shows the distribution of QT risk scores according to the type of dosing regimen. Of the 154 high-risk alerts, 82 (53%) would have been triggered by routine medications alone (Figure 5B, opaque bars), meaning that the remaining 72 alerts (47%) were attributable to the contribution of PRN drugs. 27 alerts (17%) would have been triggered by as-needed (PRN) drugs alone (Figure 5C, opaque bars). Among the latter, 89 (58%) had a QT risk score between 1 and 3, which, although insufficient to trigger alerts individually, contributed to the cumulative score across the full prescription (Figure 5C, light bars). Furthermore, when considering only as-needed drugs that were administered within the 24 h preceding an alert, only 2 prescriptions (1.3%) were associated with a high risk of QT prolongation (Figure 5D, opaque bars), and 46 (30%) alerts had a risk score between 1 and 3. This showed that the systematic inclusion of all PRN drugs, regardless of recent administration, nearly doubled the number of high-risk alerts compared to considering routine medications alone.

### 3.4 Risk factors and potential complication in patients with QT prolongation

As expected, patients in the geriatric ward were older, with a mean age of 85.9 years compared to 74.7 years in the orthopedic ward. They also exhibited a higher prevalence of all other QT prolongation risk factors considered in the study with a higher average number of risk factors (3.1 versus 2.1) (Table 2). In addition, nearly all geriatric patients (93.5%) had at least one additional risk factor besides age over 65. Regarding complications potentially associated with QT prolongation, no cases of TdP, cardiac arrest, or syncope due to cardiac arrhythmia were identified during the hospital stay following a high-risk QT alert.

**TABLE 2:** Risk factors for QT prolongation.

Risk factor	Geriatrics n (%)	Orthopedics n (%)
Age >65 years	123 (100)	23 (74.2)
Female sex	73 (59.3)	18 (58.1)
Impaired kidney function	52 (42.3)	8 (25.8)
Impaired liver function	9 (7.3)	1 (3.2)
Bradycardia	13 (10.6)	0 (0)
Cardiovascular disease	46 (37.4)	3 (9.7)
Hypothyroidism	21 (17.1)	1 (3.2)
Hypocalcemia (<2.2 mmol/L)	18 (14.6)	2 (6.5)
Hypokalemia (<3.6 mmol/L)	18 (14.6)	1 (3.2)
Hypomagnesemia (<0.59 mmol/L)	6 (4.9)	0 (0)
Average number of risk factors	3.1	2.1
Number of risk factors		
0	0 (0)	2 (6.5)
1	8 (6.5)	5 (16.1)
2	28 (22.8)	13 (41.9)
3	44 (35.8)	9 (29.0)
4	30 (24.4)	2 (6.5)
5	9 (7.3)	0 (0)
6	4 (3.3)	0 (0)

The percentages of QT, long risk factors refer to proportions calculated from the 154 patient records that were manually reviewed, rather than from the entire extracted dataset.

## 4 Discussion

Clinical intervention rates (defined as ECG ordering and/or treatment modification) varied between geriatric and orthopedic patients, ranging from 60.2% to 91.9% in geriatrics (depending on the 1-day and 7-day thresholds), and from 45.2% to 80.6% in orthopedics. The intervention rates predominantly reflected ECG performance rather than therapeutic adjustments. Only 3 of 154 patients (2.0%) had their QT-prolonging medications modified following an alert, all in the geriatric ward. This means that treatment modification contributed minimally to the composite primary outcome. However, although the absolute number of treatment modifications remained low, it is important to note that these three cases involved manifest QT prolongation (506–563 m). These high-risk situations were directly identified through ECGs performed following an alert. While the overall intervention rate is modest, the detection and subsequent mitigation of these three potentially life-threatening risks highlight the clinical utility of the system as a sentinel for critical cases. Nevertheless, the low rate of medication changes is particularly noteworthy given that many alerts involved drugs with safer alternatives (as discussed below), suggesting missed opportunities for proactive risk reduction through prescribing optimization. The higher intervention rate in geriatrics may reflect greater awareness of adverse drug reactions, given the higher prevalence of frailty and polypharmacy in this population. While prior studies, such as the one by Van der Sijs et al. (2009), reported ECG follow-up rates as low as 25% within a week after a DDI alert in non-intensive care unit wards, our observed rates may suggest better responsiveness. However, since it is unclear whether these ECGs were directly triggered by alerts and considering that guidelines recommend ECG monitoring for any patient receiving at least one high-risk QT-prolonging drug or certain combinations involving lower-risk agents, current practices in both medical wards still fall short of recommended standards.

Moreover, since the indication for ECGs was not always documented, it is possible that some ECGs were performed for unrelated reasons. Therefore, the actual number of ECGs ordered specifically for QT monitoring may be even lower. This highlights potential shortcomings of the current CDSS design, in which several key features are lacking. Improvements are needed to address alert fatigue and enhance clinical relevance, but more fundamentally, to improve the positive predictive value (PPV) of the alert system as a whole. As highlighted by previous work on this topic (Carli et al., 2018), the cumulative burden of low-PPV alerts compounds alert fatigue and undermines clinician trust in the entire CDSS.

Improving alert specificity is therefore essential. Incorporating real-time patient-specific data (recent ECG results, electrolyte values, dose-dependent risk) and accounting for actual drug administration, particularly excluding PRN drugs not given within the previous 24 h, could markedly improve positive predictive value while maintaining safety surveillance. In our cohort, excluding non-administered PRN drugs would have prevented nearly half of high-risk alerts. In addition, integrating actionable guidance at the point of prescribing, such as suggesting safer alternatives within the same therapeutic class, may enhance clinical relevance and reduce passive overrides. The refinement of tiered alert presentation, such as reserving interruptive alerts for higher-probability risk scenarios, could further mitigate alert fatigue.

Regarding drug distribution, alerts were mostly driven by combinations of drugs individually associated with low QT prolongation risk, which may make it more difficult for prescribers unfamiliar with pharmacodynamic interactions to recognize the overall risk. Nonetheless, certain drug classes were frequently implicated, offering possible targets for safer prescribing.

Antipsychotics were the most prescribed QT-prolonging drugs in both geriatric and orthopedic wards. While all antipsychotics block cardiac potassium channels to some extent, their QT-prolongation potential varies (Reilly et al., 2000; Glassman and Bigger, 2001). While haloperidol and levomepromazine carry a higher risk, drugs like aripiprazole, paliperidone, risperidone, quetiapine, olanzapine, and amisulpride are considered lower-risk agents (Fanoe et al., 2014; Leucht et al., 2013; Huhn et al., 2019; Manolis et al., 2019). A 2019 review by Manolis et al. recommends aripiprazole and paliperidone as first-line options in patients at QT risk (Manolis et al., 2019). Although the use of higher-risk drugs like haloperidol may be necessary in some situations, some alternatives with lower QT-prolongation potential may be considered when clinically appropriate. Also, QT risk varies depending on the clinical context and some observed associations with mortality may be influenced by indication bias, particularly in frail elderly patients suffering from delirium that may be more prone to receive haloperidol (Hulshof et al., 2015).

Antidepressants, especially escitalopram and citalopram, were also frequently implicated. Their predominance may reflect selection bias, as these two drugs are well known for their QT-prolonging potential. Although both belong to the selective serotonin reuptake inhibitors (SSRI) drug class and are first-line treatments for depression, safer alternatives within the same class such as sertraline exist (Ojero-Senard et al., 2017). Additionally (es)citalopram is metabolized via CYP2C19, exposing it to pharmacokinetic interactions, particularly with CYP2C19 inhibitors like proton pump inhibitors, which are commonly co-prescribed (Gjestad et al., 2015).

Among antiemetics, ondansetron and metoclopramide were prescribed in nearly equal proportions (40.8% and 39.5%, respectively). Given that ondansetron is associated with a known risk of TdP and metoclopramide with a conditional risk (Baysari et al., 2017), the latter should be preferred when clinically appropriate, especially in patients with QT risk factors. However, clinicians should remain aware of its potential extrapyramidal adverse effects. Also, ondansetron remains more effective in preventing postoperative nausea and vomiting (Wu et al., 2012). Since their mechanisms of action differ, combining antiemetics may improve efficacy (Weibel et al., 2020), but may also increase QT prolongation risk. Whenever feasible, prioritizing metoclopramide could help reduce proarrhythmic risk.

Also, our findings confirm that geriatric inpatients present a significantly higher burden of QT prolongation risk factors. Beyond age, which is an inherent factor in this population, a wide range of additional risk factors were more present in the geriatric ward, such as renal failure, cardiovascular diseases, along with electrolyte imbalances such as hypokalemia and hypocalcemia. This clustering of risk factors suggests a heightened baseline vulnerability in older adults, reinforcing the relevance of QT surveillance tools in this setting.

Given that CredibleMeds is widely used as a reference for the classification of QT-prolonging drugs, we performed a descriptive comparison between Riskbase and CredibleMeds classifications (see Supplementary Material). This comparison suggests an overall concordance between the two systems, with lower Riskbase scores (A = 1) corresponding mainly to “conditional” or “possible” risk of TdP categories, and higher scores (B = 2, C = 3) aligning more frequently with “known risk” of TdP. Notably, a non-negligible number of well-recognized QT-prolonging drugs are classified as score 2, including macrolides, fluoroquinolones, and azole antifungals, which still corresponds to a clinically relevant risk. In our dataset, only a very limited number of medications were classified as score 3 (e.g., methadone).

Regarding haloperidol, while some sources may describe its absolute QT-prolonging effect as relatively modest (Huhn et al., 2019), it is classified as “known risk” in CredibleMeds (Home), which is consistent with its classification within the Riskbase system. However, this comparison does not represent a formal validation, and differences in classification may still exist, reflecting inherent limitations of such scoring systems and their ability to capture inter-individual variability.

To further assess the clinical relevance of the Riskbase score, we conducted an exploratory analysis of measured QTc intervals across different risk levels (see Supplementary Material). A positive trend was observed, with mean QTc values being higher in the high-risk group (∼460 m) compared to the lower-risk categories (∼430 m). This correlation was particularly visible when pharmacological risk was combined with electrolyte imbalances. While these preliminary findings suggest that higher scores reflect a tangible physiological risk, the limited sample size of the lower-risk group (n = 30) precludes definitive statistical conclusions. This suggests that the low rate of treatment modification is more likely due to the complexity of managing polymorbid patients rather than a lack of accuracy in the risk scale itself.

This study has several limitations. First, it was not always possible to determine whether ECGs were ordered in direct response to alerts, as only 5 of 154 (3%) explicitly mentioned QT risk, and in 66 cases (43%) no reason was documented. The absence of a comparable control group prevented us from comparing ECG rates between patients with and without Riskbase alerts, which would have allowed a more robust assessment of the CDSS impact. Second, the CDSS did not allow documentation of alert overrides, limiting assessment of alert appropriateness. Third, the risk score included all as-needed (PRN) medications regardless of administration, potentially overestimating QT risk; incorporating only recently administered PRN drugs could reduce false positives. An additional limitation lies in the timing of the study, which was conducted during the COVID-19 pandemic, a period that may have influenced prescribing and clinical practices. Although wards dedicated to COVID-19 care were excluded, indirect effects may have influenced findings, limiting generalizability. Finally, the study population was limited to geriatric and orthopedic surgery patients. High-exposure specialties such as psychiatry and cardiology may have different prescribing practices, alert frequencies, and monitoring challenges, and should be included in future studies to better evaluate CDSS performance across diverse clinical settings.

## 5 Conclusion

Despite the availability of pharmacodynamic alerts for QT prolongation, adherence to ECG monitoring remains suboptimal, and treatment adjustments are rare (2% of cases). Our findings emphasize the need to improve alert specificity and clinical relevance by optimizing CDSS design and incorporating patient-specific factors, including timing and dosing regimens of medications. Limiting risk calculations to recently administered PRN drugs could reduce false-positive alerts without compromising safety. This study also illustrates real-world prescribing patterns and underscores opportunities to promote alternatives with lower QT-prolongation potential within common drug classes, helping clinicians to reduce QT prolongation risk when clinically appropriate.

## Data Availability

The original contributions presented in the study are included in the article/Supplementary Material, further inquiries can be directed to the corresponding author.
