# Supplementary material for: QT prolongation alerts lead to monitoring but rarely to therapeutic changes: a prospective hospital study
Source: Front Pharmacol. 2026 May 7;17:1833921. doi: 10.3389/fphar.2026.1833921 (PMC13190392; doi:10.3389/fphar.2026.1833921)
Supplement: Supplementary file 1 [file Supplementaryfile1.docx]

**Table S1. Comparison of QT prolongation risk between Riskbase and CredibleMeds databases**

| Drug | Riskbase score for long QT risk (A = 1, B = 2, C = 3) | CredibleMeds classification |
| --- | --- | --- |
| abiraterone | A | Conditional Risk |
| adenosine | B | Not specified |
| amiodarone | C | Known Risk |
| amisulpride | A | Conditional Risk |
| amitriptyline | A | Conditional Risk |
| aripiprazole | A | Possible Risk |
| azithromycin | B | Known Risk |
| carbimazole | A | Not specified |
| cimetidine | A | Conditional Risk |
| cinacalcet | A | Not specified |
| ciprofloxacin | B | Known Risk |
| citalopram | B | Known Risk |
|  |  |  |
| clarithromycin | B | Known Risk |
| clomipramine | A | Conditional Risk |
| clotiapine | A | Possible Risk |
| clozapine | A | Possible Risk |
| degarelix | A | Possible Risk |
| domperidone | B | Known Risk |
| donepezil | B | Known Risk |
| droperidol | B | Known Risk |
| enzalutamide | A | Not specified |
| erythromycin | B | Known Risk |
| escitalopram | B | Known Risk |
| famotidine | A | Conditional Risk |
| flecainide | C | Known Risk |
| fluconazole | B | Known Risk |
| fluoxetine | A | Conditional Risk |
| flupentixol | A | Possible Risk |
| formoterol | A | Special Risk |
| galantamine | A | Conditional Risk |
| haloperidol | B | Known Risk |
| heparin | A | Not specified |
| hydroxychloroquine | B | Known Risk |
| hydroxyzine | B | Conditional Risk |
| indacaterol | A | Special Risk |
| indapamide | A | Conditional Risk |
| ivabradine | A | Known Risk |
| levofloxacin | B | Known Risk |
| levomepromazine | B | Known Risk |
| lithium | A | Possible Risk |
| loperamide | A | Conditional Risk |
| melitracen | A | Not specified |
| memantine | A | Not specified |
| methadone | C | Known Risk |
| metoclopramide | A | Conditional Risk |
| metronidazole | A | Conditional Risk |
| mianserin | A | Possible Risk |
| moxifloxacin | C | Known Risk |
| norfloxacin | A | Possible Risk |
| ofloxacin | B | Possible Risk |
| olanzapine | A | Conditional Risk |
| olodaterol | A | Special Risk |
| ondansetron | B | Known Risk |
| oxycodone | A | Not specified |
| paroxetine | A | Conditional Risk |
| pipamperone | A | Possible Risk |
| quetiapine | A | Conditional Risk |
| quinine | B | Conditional Risk |
| ranolazine | A | Conditional Risk |
| risperidone | A | Conditional Risk |
| rivastigmine | A | Possible Risk |
| salbutamol | A | Special Risk |
| salmeterol | A | Special Risk |
| sertraline | A | Conditional Risk |
| solifenacin | A | Conditional Risk |
| sotalol | C | Known Risk |
| sulfamethoxazole | A | Special Risk |
| tizanidine | A | Possible Risk |
| trazodone | A | Conditional Risk |
| trimethoprim | A | Special Risk |
| trimipramine | A | Possible Risk |
| venlafaxine | A | Possible Risk |
| vilanterol | A | Special Risk |
| voriconazole | B | Conditional Risk |
| xylometazoline | A | Special Risk |

**Figure S2. Exploratory analysis of measured QTc intervals across different risk levels**

An exploratory analysis between the clinical decision support system risk levels and actual physiological QTc intervals was performed. While the main study focused on high-risk alerts (n=154), a random sample of 30 additional patients with lower risk scores was included for comparison. This allowed for a balanced exploratory comparison between "low-to-moderate risk" and "high-risk" populations. Results are showed using boxplots of QTc values for patients according to alert risk levels. **Due to the limited sample size, no formal statistical testing was performed. This analysis is intended to be purely exploratory and descriptive.**

**Subgroup analysis**:

- A: All patients included in the sub-analysis without further distinction.
- B: Patients with no documented electrolyte disturbances.
- C: Patients presenting with at least one electrolyte disturbance.

Definition for electrolyte disturbances:

1. Hypocalcemia (< 2.2 mmol/l)
2. Hypokalemia (< 3.6 mmol/l)
3. Hypomagnesemia (< 0.59 mmol/l)


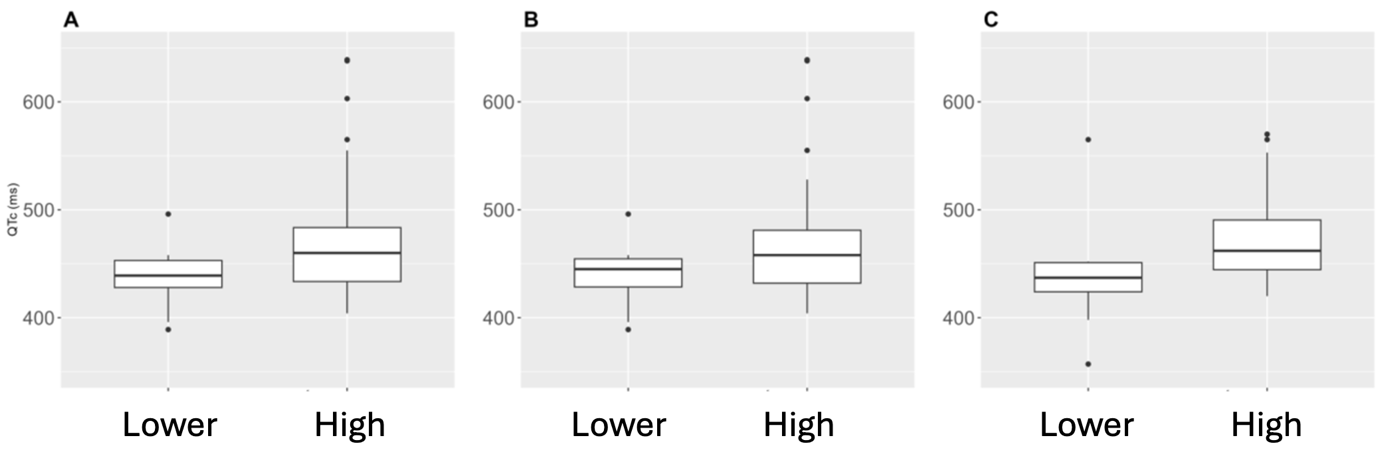


**X-axis** = level of QT prolongation risk:

- “Lower” = non-high-risk (low-to-moderate risk).
- “High” = high-risk
