# Supplementary figures and images for: QT prolongation alerts lead to monitoring but rarely to therapeutic changes: a prospective hospital study
Source: Front Pharmacol. 2026 May 7;17:1833921. doi: 10.3389/fphar.2026.1833921 (PMC13190392; doi:10.3389/fphar.2026.1833921)

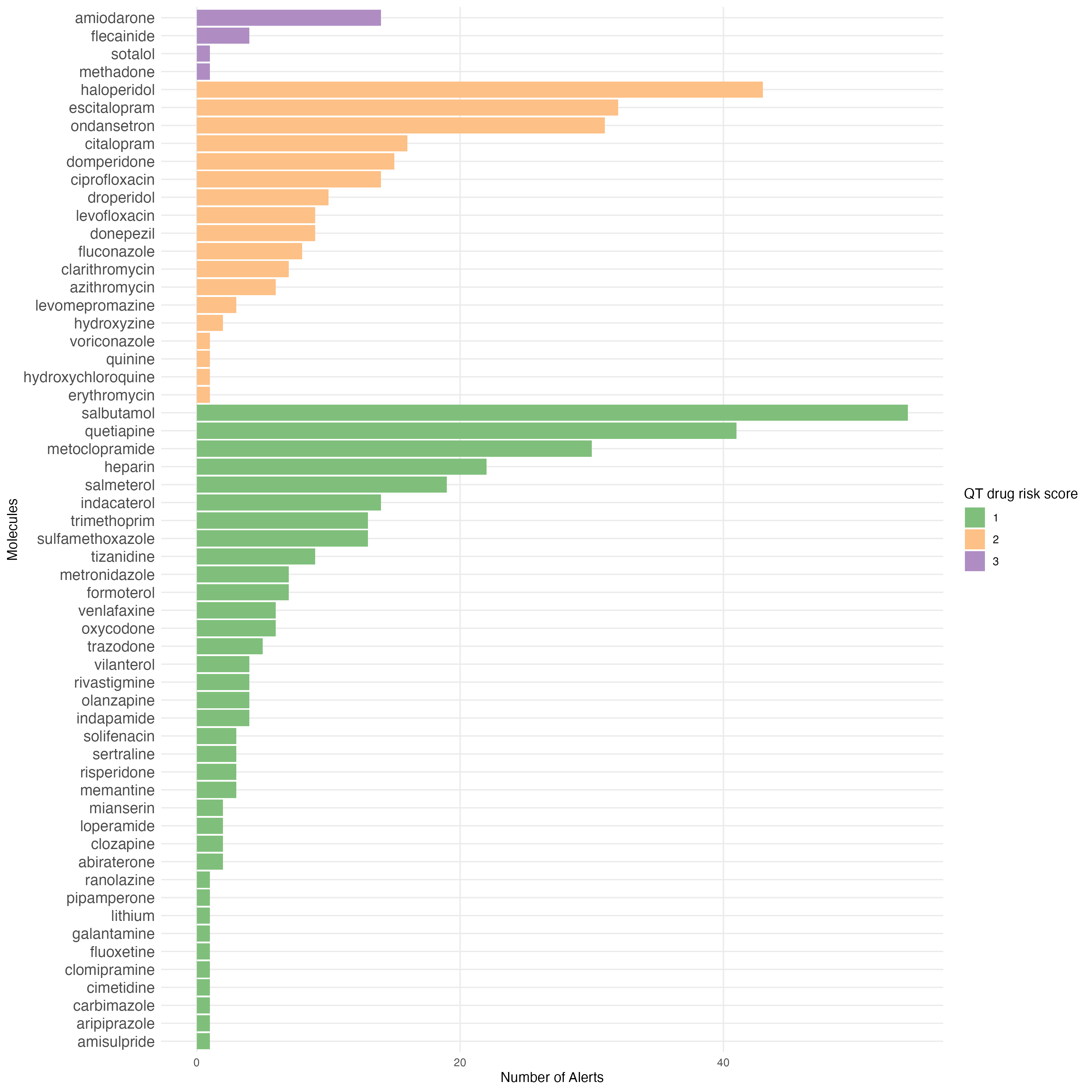

Supplement: Supplementary file 2 [file Image1.png]
